# The New Pharmacopœia

**Published:** 1914-12-19

**Authors:** 


### THE NEW PHARMACOPŒIA.

The publication of a new edition of the British Pharmacopœia is a national event. Obviously it is for the welfare of the community that prescriptions shall be dispensed in harmony with the intentions of the prescriber, and that the terms in which these intentions are expressed shall have one and the same meaning to every individual pharmacist. To the same end also it is necessary that medicines shall have uniform standards of strength, and that they shall be free from impurities and adulterants. These purposes can only be attained by the existence of an authoritative volume in which the nature, quality, and quantitative values of medicines are defined; such volume, too, must have the force of law in order that it may be binding on every citizen. Hence the General Medical Council is directed by Act of Parliament to issue the Pharmacopœia, and the definitions contained in this volume have the force of a legal enactment. Further, as new medicines come into use definitions and standards of these, too, are needed in the public interest, and new tests for efficiency and purity are devised. Therefore, at more or less frequent intervals, the Pharmacopœia must be revised and re-issued in an amended form. Such a revision has just been completed. The new edition succeeds the Pharmacopœia of 1898, and its provisions come into force on January 1, 1915.

To the production of this new issue an enormous amount of careful work has been necessary, and the experts concerned in the task have been busy with it for years. We fear the task is a somewhat thankless one. None the less, the matter is an important one, for without it the prescribing and dispensing of medicines would be thrown into hopeless confusion, and no patient could be certain that the medicine he received from the pharmacist was exactly that intended by the physician. The pharmacist would be deprived of all authoritative standards of interpretation, and would thus be free to apply to the prescription his own individual judgment and construction. If we are protected from these risks we owe our security to the makers of the Pharmacopœia. These must try to be content with the consciousness of good work that has been well done.

To the practising physician a new edition of the Pharmacopœia has a comparatively narrow importance. The one point at which it touches him is where it fixes new values for old terms. The language of the Pharmacopœia is binding on all—prescriber, dispenser, and public. Hence on and after January 1, 1915, any term used in the new edition must have the meaning attached to it in this edition. The Pharmacopœia of 1898 ceases to be, and if the same term is used in the two editions with different meanings, it is the meaning of the new edition which the dispenser must select. Take, for example, the phrase, "Tinctura Nucis Vomicæ." Under the 1898 regime this meant an alcoholic fluid containing 0.25 per cent. of strychnine; it now means an alcoholic fluid containing only one-half this percentage of strychnine. Tincture of colchicum has suffered a similar transformation; while, on the other hand, tincture of opium is increased in strength to the extent of one-third, and tincture of strophanthus is no less than four times as strong as the preparation bearing the same name in the Pharmacopœia of 1898. Obviously these alterations—and there are a number of others—have an important bearing on practical prescribing, and physicians engaged in the construction of prescriptions must, as loyal citizens, give heed to them. They may criticise them if they will, but they must perforce obey them. We do not imagine that many of them will conscientiously study the pages of the new edition, nor, indeed, is this necessary, seeing that various enterprising firms issue short summaries of the new developments; and with one or the other of such summaries an early acquaintance should be cultivated.

On one point of detail we must express regret, namely, that the Pharmacopœia, in including new drugs, has not been content with old names. Formamine, for example, was fairly well known—why, then, should it now be christened Hexamine; and similarly, why should Malourea become Barbitonum? On the whole, however, we would cultivate a grateful, if not an enthusiastic, recognition of a laborious task undertaken in the interests of a public which remains profoundly unconscious of its benefactors.

